# Fairness in Music Recommender Systems: A Stakeholder-Centered Mini Review

**DOI:** 10.3389/fdata.2022.913608

**Published:** 2022-07-22

**Authors:** Karlijn Dinnissen, Christine Bauer

**Affiliations:** Department of Information and Computing Sciences, Utrecht University, Utrecht, Netherlands

**Keywords:** bias mitigation, fairness, music recommendation systems, stakeholders, literature review

## Abstract

The performance of recommender systems highly impacts both music streaming platform users and the artists providing music. As fairness is a fundamental value of human life, there is increasing pressure for these algorithmic decision-making processes to be fair as well. However, many factors make recommender systems prone to biases, resulting in unfair outcomes. Furthermore, several stakeholders are involved, who may all have distinct needs requiring different fairness considerations. While there is an increasing interest in research on recommender system fairness in general, the music domain has received relatively little attention. This mini review, therefore, outlines current literature on music recommender system fairness from the perspective of each relevant stakeholder and the stakeholders combined. For instance, various works address gender fairness: one line of research compares differences in recommendation quality across user gender groups, and another line focuses on the imbalanced representation of artist gender in the recommendations. In addition to gender, popularity bias is frequently addressed; yet, primarily from the user perspective and rarely addressing how it impacts the representation of artists. Overall, this narrative literature review shows that the large majority of works analyze the current situation of fairness in music recommender systems, whereas only a few works propose approaches to improve it. This is, thus, a promising direction for future research.

## 1. Introduction

The art of music recommendation was traditionally performed exclusively by people, such as DJs, record store owners, and friends. In the last few decades, however, this task has been partially automated using machine learning (ML) techniques; recommender systems (RSs) in particular (Celma, 2010b). Learning from large-scale user behavior and music features, so-called music recommender systems (MRSs) can automatically produce recommendations tailored to a specific user (Ekstrand et al., 2022). This is one of the reasons why music streaming platforms, that typically integrate MRSs, have become one of the main sources of music consumption (IFPI, 2020). Consequently, the performance of MRSs highly impacts users' overall music listening experience (Lee et al., 2019) and considerably impacts artists in terms of exposure and resulting royalty payments (Ferraro et al., 2021b).

ML system users frequently perceive RS decisions as objective (Helberger et al., 2020). However, many factors make such systems' processes prone to biases, resulting in unfair outcomes (Ekstrand et al., 2022). One such factor is that ML models are created and trained by humans whose intrinsic biases may be carried over. Furthermore, the data that is used to train ML models may contain biases as well. This is problematic, as fairness is a fundamental value of human life (Folger and Cropanzano, 1998; Tyler and Smith, 1998). Moreover, anti-discrimination regulations explicitly prohibit that characteristics such as gender, age, and nationality cause different outcomes for otherwise similar people (Civil Rights Act, 1964; Age Discrimination in Employment Act, 1967; European Union, 2010, Art. 21). It is, therefore, crucial to critically review MRSs for any form of unfairness to ensure that they do not unfairly disadvantage any user or artist.

Overall, there is an increasing interest in research on fairness in ML in general (Hutchinson and Mitchell, 2019), and in RSs in particular (Ekstrand et al., 2019). One of the challenges in fairness research is that it is scattered across several disciplines (Holstein et al., 2019; Selbst et al., 2019). Moreover, it concerns several stakeholders with distinct fairness needs, calling for various bias mitigation strategies (Ekstrand et al., 2022). Considering those needs is, thus, key to both, understanding fairness in music recommendation algorithms and designing strategies to improve it. To the best of our knowledge, an overview of such needs and strategies does not yet exist for the music recommendation field specifically. Therefore, this work addresses the following research question: *What is the state-of-the-art of MRS fairness research from the various stakeholders' perspectives?* To address this RQ, we conduct a narrative literature review, giving a thorough overview of works that *explicitly* target *RS fairness in the music domain*. We also include some works that are not explicitly concerned with fairness, yet address fairness as a side effect.

In Section 2, we first define each relevant stakeholder group. Then, in the Sections 2.1, 2.2, and 2.3, we present our narrative literature review in which we address each of the relevant stakeholders separately. In Section 3, we conclude this work with a discussion of the lessons learned from this overview and derive research gaps, thereby forming a solid basis for future research.

## 2. Fairness for Multiple Stakeholders in Music Recommender Systems

The digital music value chain embraces a wide set of stakeholders, who have different goals and interests regarding the music being recommended (Bauer and Zangerle, 2019). Recommender systems literature typically distinguishes three stakeholders: platform users (end consumers), item providers, and the platform itself (Abdollahpouri et al., 2017b; Burke, 2017; Sonboli et al., 2021). Some variations can be found in literature; for instance, Mehrotra et al. (2018) and Patro et al. (2020) only consider user and item provider as stakeholders, yet not the platform; conversely, Jannach and Bauer (2020) include society at large as a fourth stakeholder.

In MRSs, there are three main stakeholders. Firstly, the **users** (Section 2.1)—also called consumers or customers—are the party consuming the music recommendations. A user may be an individual or a group of individuals, served by music streaming platforms. As individuals have different profiles containing, for instance, different characteristics, preferences, or needs, MRSs might create a better experience for some user groups than for others. Ideally, a MRS creates a good user experience for all users.

Secondly, the **item providers** (Section 2.2)—also referred to as producers or suppliers—form the stakeholder supplying the recommended music and benefiting from it being consumed or purchased. In MRS research, the artists (including performers, music producers, and songwriters) are typically the item providers, but record companies or publishers representing several artists may also be considered item providers. Each item provider usually represents a multitude of items in the form of music tracks. A higher MRS ranking for an item implies a higher chance of exposure to users, resulting in a higher chance that users interact with the item (Biega et al., 2018; Diaz et al., 2020). This is desirable, as item interaction results in revenue (Deldjoo et al., 2021). Typically, item providers have little control over when and to whom their items are recommended (Burke, 2017; Ferraro et al., 2021b).

Thirdly, the **platform** exists at the center of the music recommender ecosystem (Abdollahpouri and Essinger, 2017; Smets et al., 2022). Music streaming platforms (such as Apple Music, Deezer, Pandora, QQ Music, Spotify, and Tidal) act as an interface between huge repositories of music tracks and millions of music consumers. On such platforms, the interaction between users and items is facilitated by a MRS. A platform needs to attract and retain both users as well as item providers and, thus, benefits from a successful match between users and items (Burke, 2017). As the platforms are in control of the MRS they embed (Bauer and Zangerle, 2018) and can even significantly influence consumption decisions through functionalities such as curated playlists (Aguiar and Waldfogel, 2021), they are typically not considered being at risk of unfair treatment. Rather, platforms might impose fairness constraints to satisfy an organizational mission or meet demands of, e.g., government regulators or interest groups (Ekstrand et al., 2022). Further, there is increasing external pressure to make these platforms and their integrated MRSs fairer (Burke et al., 2018; Bauer and Zangerle, 2019; Patro et al., 2020; Ferraro et al., 2021b; Melchiorre et al., 2021).

As multiple stakeholders with possibly diverging interests are involved and affected by MRSs, **multi-stakeholder** research (Section 2.3) addresses several stakeholder groups simultaneously. Each stakeholder may have distinct fairness needs, which may further differ per context and application (Burke, 2017; Ekstrand and Kluver, 2021). Consequently, solely optimizing RSs on metrics such as user satisfaction may be detrimental to user fairness, item provider fairness, or both (Bauer and Zangerle, 2019; Patro et al., 2020). Hence, several studies urge to consider the interests of all stakeholder groups (Burke, 2017; Mehrotra et al., 2018, 2020). We note that research that addresses fairness, for example, for item providers, while also measuring performance indicators such as user satisfaction in the evaluation, are not necessarily multi-stakeholder approaches; a multi-stakeholder perspective integrates the various stakeholders fundamentally.

Table 1 provides an overview of the papers on fairness in MRSs considered in this narrative literature review. It also includes information on the research focus, methodology, considered fairness attributes, the stakeholders in the loop, and the datasets used for conducting the research.

**Table 1:** Overview of literature on fairness in music recommender systems.

**References**	**Improvement focus**	**Methodology**	**Topic**	**Considered fairness attribute(s)**	**Stakeholder focus**	**Dataset source**
Bauer et al. (2017)		Conceptual, interview	Negative impact for non-superstar artists	Popularity	Item provider	–
Bauer and Schedl (2018)	x	Data analysis, offline experiment	Improving accuracy by considering mainstreaminess and country	User country, user “mainstreaminess”	User	LFM-1b
Bauer and Schedl (2019)	x	Data analysis, offline experiment	Improving accuracy by considering mainstreaminess and country	User country, user “mainstreaminess”	User	LFM-1b
Boratto et al. (2022)	x (reproduction)	Systematic literature review, reproduction	Reproducing and comparing unfairness mitigation strategies	User age, user gender	User	LFM-1K
Celma (2010b)		Data analysis	Promotion of niche items	Popularity	User	Proprietary (Last.fm and MySpace)
Celma and Cano (2008)		Data analysis, offline experiment	Investigating popularity bias in collaborative filtering	Popularity	User	Proprietary (Last.fm)
Ekstrand et al. (2018)		Data analysis, offline experiment	Recommender effectiveness across demographics and popularity levels	Popularity, user age, user gender	User	LFM-1K, LFM-360K
Epps-Darling et al. (2020)		Data analysis	Analysis of gender distribution across popularity levels	Artist gender, popularity	Item provider	Proprietary (Spotify)
Ferraro et al. (2020)		Offline experiment	Evaluating influence of recommendation bias on artist exposure	Contemporaneity, country, gender, type (all artist attributes)	Item provider	LFM-360K
Ferraro et al. (2021a)	x	Interviews, data analysis, offline experiment, long-term simulation	Improving gender fairness	Artist gender	Item provider	LFM-360K, LFM-1b
Ferraro et al. (2021b)		Interviews	Impact of recommender systems on artists	Age, contemporaneity, country, diversity, gender, popularity (all artist attributes)	Item provider	–
Flexer et al. (2018)		Data analysis	Hubness as a technical algorithmic bias in high dimensional machine learning	^−^ ^*a*^	User, item provider	Proprietary (FM4 SoundPark)
Htun et al. (2021)		User study	Perception of fairness per user personality type	^−^ ^*b*^	User	–
Kowald et al. (2021)		Data analysis, offline experiment	Characteristics of niche music and music listeners	User “mainstreaminess”	User	LFM-1b
Kowald et al. (2020)		Data analysis, offline experiment	Investigating the impact of popularity bias on niche items, and users favoring those items	Popularity, user “mainstreaminess”	User	LFM-1b
Lesota et al. (2021)		Data analysis, offline experiment	Effect of popularity bias per gender	Popularity, user gender	User	LFM-2b
Mehrotra et al. (2018)	x	Offline experiment	Relevance, fairness and satisfaction trade-off in a two-sided marketplace	Popularity	User, item provider	Proprietary (Spotify)
Mehrotra et al. (2020)	x	Offline experiment	Contextual bandits that consider multiple objectives (e.g., gender diversity, niche items)	Artist gender, popularity	User, item provider	Proprietary (Spotify), Simulated data
Melchiorre et al. (2021)	x	Data analysis, offline experiment	Improvement of gender fairness considering popularity bias	User gender	User	LFM-2b
Mousavifar and Vassileva (2022)		User study	Using explanations to increase user satisfaction with fair recommendation	Popularity	User, item provider	–
Neophytou et al. (2022)		Offline experiment, reproduction	Reproducing recommendation utility for different user groups	Popularity, user age, user country, user gender	User	LFM-360K
Oliveira et al. (2017)	x	Offline experiment	Considering diversification and user preferences simultaneously in a multi-objective approach	Contemporaneity, gender, genre, locality (all artist attributes)	User, item provider	LFM-1b, Simulated data
Schedl and Bauer (2017)		Offline experiment	Improving accuracy by considering mainstreaminess	User “mainstreaminess”	User	LFM-1b
Shakespeare et al. (2020)		Data analysis, offline experiment	Investigating gender fairness	Artist gender	Item provider	LFM-360K, LFM-1b, Simulated data

a *Hubness can create unfairness for any attribute*.

b *Not transparent which fairness attributes participants were considering*.

### 2.1. User Perspective

From the user perspective, fairness in MRS is primarily studied based on distinct user groups defined by personal characteristics. In addition to groups based on protected characteristics, groups differentiated by other characteristics may experience unfairness as well.

A wealth of literature analyzes popularity bias and subsequent mitigation strategies in various application domains (e.g., Figueiredo et al., 2014; Abdollahpouri et al., 2017a; Wei et al., 2021). It is, for instance, widely acknowledged that collaborative filtering-based recommendation approaches are prone to popularity bias (Celma and Cano, 2008; Jannach et al., 2015). The music domain is a well-known example of the long-tail economy (Anderson, 2006) and popularity bias is, thus, particularly relevant. It can be considered either a problem (Anderson, 2006) or a desired feature as popularity in the community signifies some relevancy (Celma, 2010b). In general, many works address popularity bias in MRSs with various intentions. Some address the cold-start problem for items without prior user ratings to make them recommendable (e.g., Ferraro, 2019); others aim at increasing user satisfaction by adding novelty through recommending items from the long tail (e.g., Bedi et al., 2014); yet other works leverage the long tail to specifically address discovery (e.g., Domingues et al., 2013). While fairness is not always necessarily put in the loop of the investigation, this research thread does address fairness aspects.

As for insights from works that explicitly consider user fairness in MRSs, recommendation accuracy tends to be higher for “mainstream” users, who are inclined toward what is popular, compared to “beyond-mainstream” users who prefer less popular items (Kowald et al., 2020, 2021). This also holds when defining user groups based on a more fine-grained music taste level (Schedl and Bauer, 2017; Kowald et al., 2021). Some works (e.g., Bauer and Schedl, 2019) have proposed mechanisms that better reflect the preferences of beyond-mainstream users.

When defining user groups based on user country, popularity bias also negatively affects MRS performance for groups from countries with preferences beyond the global mainstream (Bauer and Schedl, 2018; Neophytou et al., 2022). In a later work, Bauer and Schedl (2019) propose context-prefiltering approaches to mitigate this issue. Zooming in on another user characteristic, several studies investigate gender. They show that popularity bias particularly affects minority gender groups (in these studies: women), resulting in lower-quality recommendations in terms of accuracy and coverage (e.g., Lesota et al., 2021; Melchiorre et al., 2021). In addition to finding similar results for user gender, Ekstrand et al. (2018) and its reproducibility study by Neophytou et al. (2022) found performance differences for different user age groups, too. Here, the older user group received lower-quality recommendations.

Lastly, on the mitigation side, Boratto et al. (2022) present a reproducibility study focusing on user age and gender, applying various mitigation strategies in the music and movie domains. Different from the movie domain, the size of the user group was not indicative of the recommender accuracy in the music domain. Given their indecisive results, it is important to look beyond popularity bias and demographic group size to understand the drivers of demographic differences.

Melchiorre et al. (2020) define user groups based on personality traits. In contrast to the work on gender, age, and country, personality traits are not among the characteristics acknowledged by anti-discrimination regulations, and fairness research is also not clear about this issue either. Nonetheless, they may be a source of bias and an opportunity for MRS improvement. Melchiorre et al. (2020) illustrate this by showing that scoring low on the personality traits openness, extraversion, and conscientiousness results in higher recommender performance, whereas scoring low on neuroticism or agreeableness leads to lower performance. Additionally, Htun et al. (2021) study the effect of personality traits on the perception of fairness in group recommendations when creating group music playlists. Here, the personality trait openness is negatively correlated with the perception that fairness is important in groups. Given that diversity needs and personality traits correlate (Chen et al., 2013), considering those traits in user modeling may help improve MRS performance.

### 2.2. Item Provider Perspective

When considering harm against music providers caused by unfairness in MRSs, research mainly focuses on group fairness (Singh and Joachims, 2018). Item provider groups in MRS research have been primarily defined based on gender (Ekstrand and Kluver, 2021; Ferraro et al., 2021a). Several approaches are used to study and mitigate item provider gender bias, illustrating that a multifaceted approach is needed. To date, most research has focused on understanding existing gender biases (e.g., Wang and Horvát, 2019; Epps-Darling et al., 2020). The former analyzed a Spotify streaming sample and found a disparity between artist genders in users' listening behavior. In “organic” streaming, such as streams originating from a user library or user's search, 21.75% of tracks were from either a woman or multi-gender formation. For streams programmed by MRSs, this number was 23.55%. This gender gap in listening behavior is further reflected in commonly used datasets such as LFM-1b and LFM-360k, in which 23% of (solo) artists are women (Ferraro et al., 2021a). These datasets roughly reflect the gender gap in business reality (Youngs, 2019; Epps-Darling et al., 2020). Overall, these percentages reflect the barriers to entry, and subsequently climbing to the top, for minority genders. In addition, pre-existing gender biases might influence which tracks users select in a MRS. Ferraro et al. (2020) and Shakespeare et al. (2020) found that collaborative filtering algorithms could propagate or even amplify those biases in a MRS, thereby negatively impacting minority genders. In the latter, no evidence was found for the algorithms introducing new gender biases, which is supported by Epps-Darling et al. (2020) who found that recommendation-based streaming even contained a slightly higher proportion of tracks by women than in organic listening. On the gender bias mitigation side, re-ranking is a promising method. Ferraro et al. (2021a) demonstrate breaking bias amplification through gradually increasing exposure for minority genders.

In addition to gender, Oliveira et al. (2017) consider genre, locality, and contemporaneity. Embracing these attributes, they introduce a multi-objective approach to diversification that addresses fairness for users and item providers alike. Ferraro et al. (2020) use similar categories and add artist type (e.g., solo artist, band). Their analysis of the locality attribute indicates that group size may foster exposure: the artists from the most represented countries in the dataset (here: United Kingdom and United States) reached high exposure, while minority countries were penalized.

Defining item provider groups based on their popularity level has been investigated, too (Celma and Cano, 2008; Bauer et al., 2017). Although popularity bias is a frequently researched topic, fairness goals are predominantly defined for MRS users and not item providers. One exception to this is Flexer et al. (2018) who study the “hubness” phenomenon, which can occur in content-based RS models that use song similarity as their main feature. Hubness refers to some music tracks being connected to many other tracks in the database without a clear semantic musical connection. This may introduce unfairness for tracks that are more similar semantically, but not recommended as often.

To date, one study directly discusses fairness in MRSs with the item providers themselves: Ferraro et al. (2021b) interviewed artists about their perception of fairness in MRSs, and how item provider fairness could be improved on music streaming platforms. In those interviews, the main noted fairness improvement areas relate to nurturing diversity in general, and in particular to gender representation, addressing popularity bias, and providing a better representation of genres beyond the mainstream. These topics also correspond to the aforementioned research focuses in literature.

### 2.3. Multi-Stakeholder Perspective

Studies may simultaneously take several different MRS stakeholder objectives (e.g., satisfaction, utility, fairness, or diversity) into account. Generally, across application domains, a trade-off between such objectives is reported (Cramer et al., 2018; Mehrotra et al., 2018; Singh and Joachims, 2018), though it is possible that multi-stakeholder objective optimization benefits all stakeholders. Item provider fairness, for example, does not have to be detrimental to user satisfaction (Mehrotra et al., 2018), and persuasive strategies may even be implemented to promote new and less popular artists while increasing user satisfaction (Mousavifar and Vassileva, 2022). Furthermore, even if users do not directly benefit from or even consider fairness for item providers, they indicate that it is important to incorporate it in RSs (Sonboli et al., 2021).

Overall, fairness-related multi-stakeholder MRS work mainly defines objectives and stakeholders rather than aiming to improve fairness. Mehrotra et al. (2018), though, do contribute to fairness improvement by introducing a counterfactual estimation framework that balances provider fairness with user relevance and can optimize either, aiming to provide an alternative for expensive online A/B tests. In another study, Mehrotra et al. (2020) use “contextual bandits” that can optimize multiple objectives simultaneously in a fair way, this time focusing on user- and platform objectives as opposed to item providers.

We might also draw inspiration from multi-stakeholder MRS research where fairness is not an explicitly defined goal. For instance, Unger et al. (2021) introduce a multi-objective RS that aims to fulfill both user satisfaction (measured by saves, likes, and engagement) and item provider satisfaction (determined by, e.g., acquiring new fans). A similar approach may be taken to implement fairness objectives for multiple stakeholders. Patro et al. (2020) propose FairRec, which exhibits fairness for both user and item provider while the loss in overall recommendation quality remains marginal. FairRec has, however, not been applied to the music domain yet.

## 3. Discussion and Conclusions

This literature overview demonstrates that, while there is increasing interest in research on fairness in RSs in general, comparatively little research has addressed the music domain. Below, we discuss the main findings we derive from this review.

### 3.1. Research Focus

Contrary to what literature frequently claims (e.g., Patro et al., 2020; Ferraro et al., 2021b), fairness in this context has been addressed from both the user perspective and the item provider perspective. Yet, multi-stakeholder approaches to fairness are scarce. This review also shows that the large majority of MRS fairness works analyzes the current situation, using existing approaches and available datasets. We, therefore, identify improvement-focused research as the main research gap. A major challenge remains here: we still need to improve our understanding of the normative nature of fairness. While an entirely fair system is likely unachievable, it is crucial to recognize RS fairness issues, mitigate them, and incrementally improve fairness over the current state.

### 3.2. Gender Bias

Interestingly, various MRS works address gender fairness, both for user and item providers. We speculate that this focus has emerged from gender being an immutable characteristic, the wide acknowledgment that gender fairness is of societal relevance, and gender labels being available to some extent in relevant datasets. While it is a known limitation that a binary concept of gender oversimplifies gender expression, current datasets predominantly restrict the gender labels to man and woman (Shakespeare et al., 2020; Ferraro et al., 2021a; Boratto et al., 2022). A notable exception is the work by Epps-Darling et al. (2020).

### 3.3. Popularity Bias

While popularity bias may be considered an item provider fairness issue as the gap between popular and unpopular items increases, research frequently focuses on the user. Addressing popularity is seen as a means to provide more diverse content to increase user satisfaction. Similarly, we observe that some works do not explicitly focus on fairness, but still demonstrate fairness intentions or improvements in their research. As this review focused on works that address fairness *explicitly*, this overview is not intended to be exhaustive.

### 3.4. Data Availability

As can be seen in Table 1, the most frequently used datasets originate from Last.fm: LFM-1b (Schedl, 2016), LFM-1K, LFM-360K (both Celma, 2010a), and the recently added LFM-2b (Schedl et al., 2022). This results in only a few datasets being used for research on fairness in MRS; most of which are either based on the same or similar Last.fm data, or are proprietary and therefore not accessible to other researchers. Overall, this means that the used datasets might not be representative. Additionally, only a few open datasets in the music domain contain user interaction or preference data. They also typically include only limited fairness-related stakeholder metadata (e.g., gender, age, ethnicity), as sensitive data is often not shared (Stoikov and Wen, 2021). For ethical reasons, it is debatable whether it should be. Lastly, a current limitation is the focus on short-term bias mitigation, while real world-systems are active over years (Shakespeare et al., 2020). Longitudinal data or simulation frameworks are needed to better address these temporary aspects and to study fairness in MRS in the long run. Summing up, to achieve significant MRSs fairness improvements, richer and more representative data is needed.

## Author Contributions

KD and CB contributed to writing and revising the manuscript draft, as well as the final submitted version.

## Conflict of Interest

The authors declare that the research was conducted in the absence of any commercial or financial relationships that could be construed as a potential conflict of interest.

## Publisher's Note

All claims expressed in this article are solely those of the authors and do not necessarily represent those of their affiliated organizations, or those of the publisher, the editors and the reviewers. Any product that may be evaluated in this article, or claim that may be made by its manufacturer, is not guaranteed or endorsed by the publisher.
